# Effects of anodal transcranial direct current stimulation over the contralesional hemisphere on motor recovery in subacute stroke patients with severe upper extremity hemiparesis: Study protocol for a randomized controlled trial

**DOI:** 10.1097/MD.0000000000019495

**Published:** 2020-04-03

**Authors:** Stephanie Hyeyoung Lee, Won-Seok Kim, Jihong Park, Junsik Kim, Nam-Jong Paik

**Affiliations:** Department of Rehabilitation Medicine, Seoul National University College of Medicine, Seoul National University Bundang Hospital, Seongnam, Republic of Korea.

**Keywords:** stroke, stroke rehabilitation, transcranial direct current stimulation, upper extremity motor impairment

## Abstract

**Introduction:**

Upper extremity motor impairment is one of the major sequelae of stroke, resulting in limitations of activities of daily living. Recently, contralesional cortical activation has been reported to be important for motor recovery in stroke patients with severe upper extremity hemiparesis due to the extensive corticospinal tract involvement. We therefore designed this study to investigate the effects of contralesional anodal transcranial direct current stimulation (tDCS), which induces cortical activation, in stroke patients with severe upper extremity motor impairment.

**Methods and analysis:**

We will recruit patients with subacute stroke (<3 months after onset) with unilateral upper extremity weakness who meet the following criteria: Shoulder Abduction and Finger Extension (SAFE) score below 8, Fugl-Meyer Assessment for upper extremity (FMA-UE) score ≤25, and absent motor evoked potential (MEP) response on the affected extensor carpi radialis muscle. Subjects will be randomly allocated to either the intervention (n = 18) or the control group (n = 18). The intervention group will undergo 10 sessions of robotic arm rehabilitation with simultaneous anodal tDCS over the contralesional premotor area, whereas the control group will receive sham tDCS during the same sessions. One daily session consists of 25 minutes.

The primary outcome measure of this study is the Fugl-Meyer Assessment score of the upper extremity; the secondary outcome measures are the Korean version of the Modified Barthel Index, the Brunnstrom stage of the affected arm and hand, the Box and Block Test, the Modified Ashworth Scale, the Manual Muscle Power Test, and the patient's encephalographic laterality index.

**Discussion:**

Findings of this study will help to establish an individualized tDCS protocol according to the stroke severity and to find out the EEG parameters to predict the better recovery in subacute stroke patients with severe upper extremity hemiparesis.

**Ethics and Dissemination:**

The study was approved by the Seoul National University Bundang Hospital Institutional Review Board (IRB No. B-1806-475-006) and will be carried out in accordance with the approved guidelines. The results of the trial will be submitted for publication in a peer-reviewed journal.

## 1 Introduction

Upper extremity motor impairment is one of the major sequelae of stroke, resulting in limitations of activities of daily living (ADL) and lowered quality of life.^[1,2]^ After stroke, damage to the ipsilesional primary motor cortex (M1) results in an imbalance between the 2 hemispheres (hypoexcitability of ipsilesional M1 and hyperexcitability of contralesional M1), with greater imbalance associated with worse outcomes.^[3]^ Transcranial direct current stimulation (tDCS) has therefore traditionally been used to reduce the imbalance between the two hemispheres, based on the “interhemispheric inhibition (IHI) model.”^[4–6]^ Anodal tDCS over ipsilesional M1 is used to directly increase excitability in ipsilesional M1, whereas cathodal tDCS over contralesional M1 is used to suppress the overexcitability of contralesional M1 and abnormally high IHI from contralesional to ipsilesional M1, which results in an increase in ipsilesional M1 excitability.^[7,8]^

However, contralesional cathodal tDCS and repetitive transcranial magnetic stimulation have yielded conflicting results on upper limb functional recovery after stroke,^[9–12]^ which might be associated with the diversity of participants’ severity of upper limb hemiparesis and chronicity after stroke. For example, patients with stroke with severe damage to the ipsilesional corticospinal tract showed poor responses to cathodal tDCS of contralesional M1, whereas patients with less damage showed good responses.^[13]^ Di Pino et al therefore suggested the “bimodal balance-recovery model,”^[5,14]^ in which the role of the activation of the contralesional hemisphere in recovery depends on the degree of corticospinal tract damage. This model postulates that IHI is less important for recovery in severe upper extremity hemiparesis, and that contralesional cortical activation induced by anodal tDCS over the contralesional hemisphere may contribute to functional recovery; this hypothesis has however not been tested in patients in a clinical trial. In addition, IHI has only been demonstrated in patients with chronic stroke.^[15,16]^ Recently, a longitudinal study in patients with stroke using paired transcranial magnetic stimulation demonstrated that an IHI imbalance in subacute stroke is not associated with worse motor recovery, which calls into question the use of cathodal tDCS over contralesional M1 in acute or subacute stroke.

Therefore, this study primarily aims to investigate the effects of anodal tDCS over the contralesional premotor cortex on motor recovery in patients with subacute stroke with severe upper extremity hemiparesis who can expect no meaningful recovery in a conventional rehabilitation setting, according to the PREP algorithm suggested by Stinear et al.^[17]^ Robotic arm rehabilitation therapy was selected as a concurrent rehabilitation modality due to its possible beneficial effects on motor recovery in severely impaired patients with stroke.^[18,19]^ Secondarily, the neurophysiological changes induced by tDCS will be investigated using electroencephalography (EEG), and used to further investigate neurophysiological substrates and markers which differentiate responders from nonresponders for this new tDCS approach.

## 2 Methods and analysis

### 2.1 Study design

This study is a double-blind, randomized, sham-controlled trial conducted at a single center. The study was approved by the Seoul National University Bundang Hospital Institutional Review Board (B-1805/468-002), and written informed consent will be provided by all participants before enrolment. The study was registered at ClinicalTrials.gov (NCT03635008).

### 2.2 Trial status

At the time of submission of this study protocol, subject recruitment is ongoing.

### 2.3 No patient and public involvement

This research was done without patient involvement. Patients were not invited to comment on the study design and were not consulted to develop patient relevant outcomes or interpret the results. Patients were not invited to contribute to the writing or editing of this document for readability or accuracy.

### 2.4 Participants

Inpatients at the rehabilitation medicine department at a single center will be screened for the study. The inclusion criteria include the following: age 18 to 85 years; first-ever stroke; ischemic or hemorrhagic stroke confirmed by magnetic resonance imaging or computed tomography; <3 months after stroke onset; and severe unilateral hemiplegic upper extremity impairment meeting the following conditions^[17]^: Shoulder Abduction and Finger Extension (SAFE) score <8, Fugl-Meyer Assessment for upper extremity (FMA-UE) ≤25, and absent motor evoked potential (MEP) response on the affected extensor carpi radialis muscle. The exclusion criteria were the following: history of brain disorders other than stroke that can cause motor deficits, such as traumatic brain injury or brain tumours; inability to follow instructions (indicated by a score ≤15 on the Korean version of the Mini-Mental State Exam^[20]^ or delirium or impaired consciousness); pregnancy; cardiac pacemakers, cochlear implants, or metals in the head (eg, clip, coil); scalp problems which might interfere with the tDCS application; poor sitting balance, poor head control, or severe upper extremity pain that could interfere with robotic arm rehabilitation; and uncontrolled severe medical conditions.

### 2.5 Recruitment

**Figure 1:**
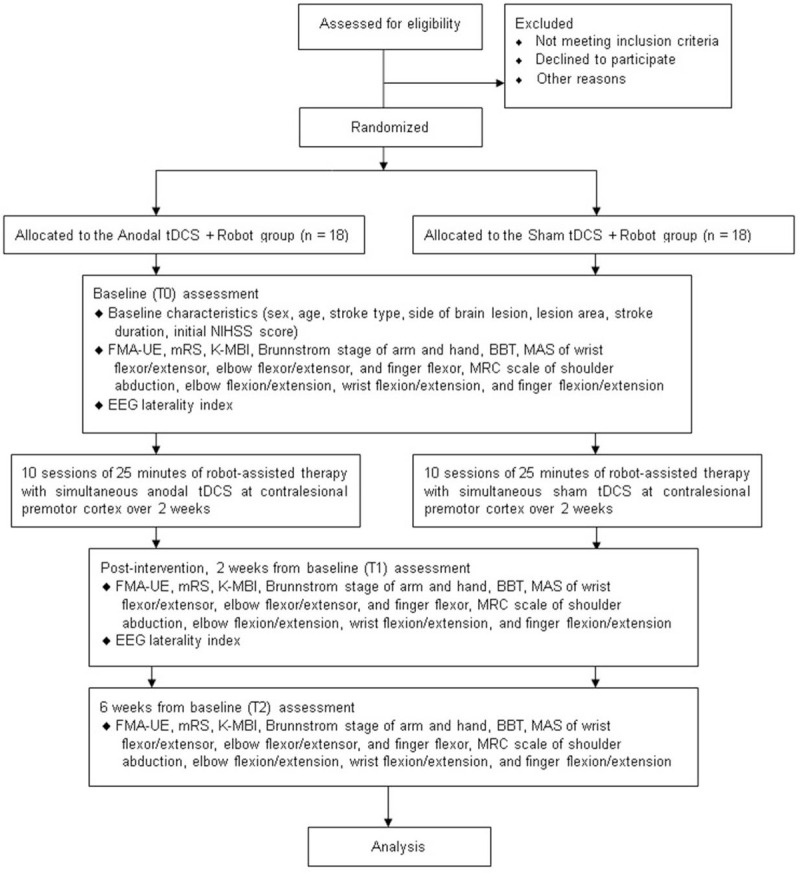
Trial design. BBT = box and block test, EEG = electroencephalography, FMA-UE = Fugl-Meyer assessment for upper extremity, K-MBI = Korean version of the modified Barthel index, MAS = modified Ashworth scale, MRC = Medical Research Council, mRS = modified Rankin Scale, NIHSS = National Institutes of Health stroke scale, tDCS = transcranial direct current stimulation.

Participants will be recruited from the inpatient rehabilitation department of Seoul National University Bundang Hospital. We will recruit 18 participants per group. The trial design is outlined in Figure 1. All potential participants will be screened for eligibility, and the principal investigator will provide them with information about the study. If patients are interested, the coinvestigators will provide additional information about the trial and obtain written informed consent from the participants. A list of the people screened and enrolled in the trial as well as reasons for ineligibility will be recorded. Recruitment commenced in July 2019.

### 2.6 Intervention

**Figure 2:**
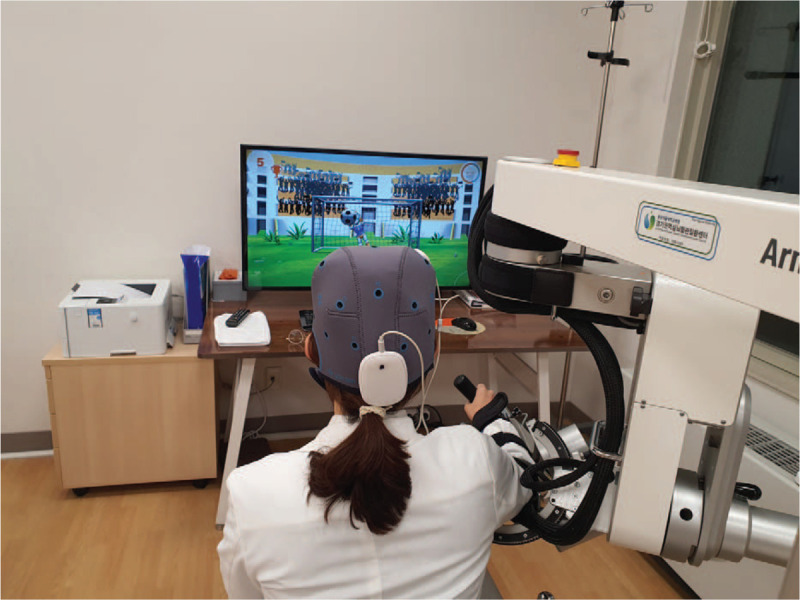
Experimental set-up.

All participants will receive 25 minutes of robot-assisted therapy with the ArmeoPower device (Hocoma, Volketswil, Switzerland), daily on weekdays for 2 weeks, for a total of 10 sessions. The robot-assisted therapy will be managed by the same experienced occupational therapist who will choose the adequate program for each participant. According to the participant's group allocation, anodal tDCS or sham tDCS will be applied concurrently with the robot-assisted therapy. The experimental setup is demonstrated in Figure 2. The time used to adjust the tDCS setup and the robotic arm will not be counted as therapy time. All subjects will undergo an additional 30 minutes of conventional occupational therapy and will be permitted to engage in physical therapy to restore gait during the intervention period. Intervention will be halted at any time, on the participant's request or on the primary investigator's decision, upon any severe adverse events.

#### 2.6.1 Anodal tDCS group

In the anodal tDCS group, anodal stimulation of the contralesional premotor cortex will be performed simultaneously with robotic-assisted therapy for 25 minutes. A constant current of 2 mA will be delivered for 25 minutes using the Ybrain tDCS System (Ybrain, Korea) via 2 saline-soaked electrodes. The anode will be positioned over the contralesional premotor cortex, which is 2.5 cm anterior to the C3 or C4 according to the international 10–20 EEG system. The cathode will be located in the ipsilesional supraorbital area.^[21]^

#### 2.6.2 Sham tDCS group

Sham tDCS will be applied with the same configurations as anodal tDCS, except for the emission of a current for only 30 seconds.

### 2.7 Assessment

Assessments will be conducted 3 times: at baseline (T0), after 2 weeks of intervention (T1), and at 4 weeks post-intervention (6 weeks post-baseline) (T2). The T0 assessment is conducted within 7 days before the start of the therapy session, the T1 assessment within 3 days after the end of the therapy session, and the T2 assessment within 7 days from day 30 after the start of the therapy session. At the T0 assessment, additional information on the participants’ baseline characteristics, including sex, age, stroke type, side of brain lesion, lesion area, stroke duration, and the initial National Institutes of Health Stroke Scale (NIHSS) score, will be collected. The NIHSS score measures neurologic deficits according to 11 categories, and the score ranges from 0 to 42, with 0 indicating no abnormality. Excellent specificity, sensitivity, and accuracy in predicting outcomes have been reported.^[22,23]^ As for the outcome measures, the primary outcome measure FMA-UE and all secondary outcome measures except the EEG laterality index (LI) (modified Rankin Scale [mRS], Korean version of the Modified Barthel Index [K-MBI], Brunnstrom stage of the arm and hand, the Box and Block Test [BBT], the Modified Ashworth Scale [MAS] of the wrist flexor/extensor, elbow flexor/extensor, and finger flexor, and the Medical Research Council [MRC] Scale of shoulder abduction, elbow flexion/extension, wrist flexion/extension, and finger flexion/extension) will be assessed 3 times. The EEG LI will be assessed twice, at T0 and at T1. Assessments last approximately 1.5 hours and regular breaks will be scheduled to mitigate fatigue and burden.

### 2.8 Outcome measures

#### 2.8.1 Primary outcome

The primary outcome is the change in FMA-UE scores between T0 and T1. The FMA-UE is a quantitative measure of motor impairment of the upper extremities, ranging from 0 to 66, with higher scores indicating less motor impairment.^[24]^

#### 2.8.2 Secondary outcomes

Secondary outcomes are the change in FMA-UE scores between T0 and T2, and the changes in mRS scores, K-MBI scores, Brunnstrom stages, BBT scores, MAS scores of the wrist flexor/extensor, elbow flexor/extensor, and finger flexor, MRC Scale scores of shoulder abduction, elbow flexion/extension, wrist flexion/extension, and finger flexion/extension between T0 and T1 and between T0 and T2, as well as the change in the EEG LI between T0 and T1.

The mRS is a categorical measure of disability incorporating mental and physical status after neurological deficits. The scale consists of 6 grades, from 0 (no symptom at all) to 5 (severe disability), and 6 (death).^[25]^ The K-MBI is the Korean version of the MBI, which measures the degree of independence of ADL according to 10 items, with the total score ranging from 0 (completely dependent) to 100 (independent).^[26]^ The Brunnstrom stage is the motor recovery stages after stroke, with the recovery process classified into six stages from 1 (flaccidity with no movements of the limbs) to 6 (individual joint movements).^[27]^ The BBT is a measure of gross manual dexterity, assessed by counting the number of blocks moved from one compartment to another across the partition in 60 seconds, with proven validity.^[28]^ The MRC is an ordinal scale of muscle power, with proven reliability, and the MAS is a scale of spasticity which is graded from 0 to 4, larger numbers indicating more increased resistance to passive movement of the joints.^[29,30]^

The EEG LI will be calculated to measure the lateralization of cortical activity during rest and task performance. LI is calculated as LI = (NL-NR)/(NL+NR), and values range between −1 and 1. An LI > 0.2 indicates a predominance of right cerebral cortical activity, an LI < −0.2 indicates predominance of left cerebral cortical activity, and an LI between −0.2 and 0.2 is considered “not lateralized.”^[31]^

Participants will wear a cap with 32 channels of sensors and detectors placed according to the 10–20 system, and signals will be recorded using the LiveAmp system actiCAP Xpress Twist (Brain Products GmbH). The total EEG task design is as follows: Rest—Task with affected hand—Rest—Task with unaffected hand. For the resting state EEG acquisition, participants will remain seated comfortably on a chair. Each resting block consists of 1 minute with eyes closed and 1 minute with eyes open, and a total of 2 blocks are recorded for 4 minutes. Hand grasping is used as the target movement for the task, and the target movement posture will be shown on an LCD monitor to guide participants. Each task block consists of 10 hand grasping trials, with grasping durations of 3 seconds and intertrial intervals of 5 seconds. The inter-block interval is 1 minute, and a total of 4 blocks will be performed.

### 2.9 Randomization and blinding

The randomized allocation to the anodal tDCS group or sham tDCS group was generated using SPSS (version 25.0). Each group consists of 18 participants with a distribution ratio of 1:1, yielding a total of 36 enrolments. According to the randomized allocation, sealed opaque envelopes with a card representing the group assignment were prepared. When a participant is being enrolled, the principal investigator opens an envelope (in numerical order). Anodal tDCS and sham tDCS modules are marked as “A” and “B.” to mask the investigator who applies the tDCS to the participants, and the principal investigator tells the investigator whether “A” or “B” will be applied. Participants, occupational therapist who conduct the robotic therapy, and researchers who carry out assessments are also blinded to the group allocation.

### 2.10 Sample size

The clinically important difference for the FMA regarding participants’ ability to move their arm has been reported as 9 to 10.^[32]^ A previous study on anodal tDCS or sham tDCS in patients with stroke revealed a standard deviation of 10.^[33]^ When estimating the drop-out rate as 10%, with a power of 80% at a 2-tailed significance level of 5%, the total required sample size is 36, that is 18 participants in each group.

### 2.11 Safety and adverse events

A Data and Safety Monitoring Plan (DSMP) is established. Adverse events and data on the participants’ physical assessments will be monitored. Major non-compliance, adverse effects caused by a medical device, and unanticipated events will be reported within 15 days. Minor noncompliance and DSMP data will be reported every 6 months.

### 2.12 Statistical analysis

The primary analysis will be done on a modified intention-to-treat (ITT) population of participants who were randomly assigned to groups and underwent >1 therapy session. Participants who complete the 10 tDCS sessions and all 3 (T0, T1, and T2) assessments per protocol will be included for the secondary endpoint analysis to verify a sustained clinical response. For between-group comparisons of baseline characteristics, Student *t* tests or Mann–Whitney *U* tests for continuous variables and *χ*^2^ tests or Fisher exact test for categorical variables, depending on normality, will be used. Student *t* tests or Mann–Whitney *U* tests will be performed to investigate if the changes in primary and secondary outcome measures are different between the anodal tDCS and sham group. All statistical analyses will be performed using SPSS (version 25.0), and *P* values <.05 will be considered statistically significant. In the repeated measures analysis including assessments of T2, Bonferroni correction will be applied and the significance level will thus be adjusted to 0.05/2 = 0.025.

## 3 Discussion

This study aims primarily to investigate the effects of anodal tDCS over the contralesional premotor cortex in patients with subacute stroke with severe upper extremity hemiparesis. Under the concept of IHI model, contralesional cathodal tDCS has been used with conflicting results. Therefore, another model called “bimodal balance-recovery model” is introduced highlighting ∼ the promotion of contralesional cortex function in stroke with severe motor impairment. This will be the first randomized controlled trial investigating the effect of contralesional anodal tDCS in patients with subacute stroke with severe upper extremity motor impairments. The expected results will be better outcome measure improvements in anodal tDCS group than sham tDCS group. Secondarily, the EEG signals are collected to further investigate neurophysiological markers to predict responders or nonresponders for this new tDCS protocol. Results of this study will help to establish a new EEG signal-based precise tDCS protocols for stroke patients with severe motor impairment.

## Acknowledgments

The authors thank Ybrain, who provided the tDCS platforms and technical support.

## Author contributions

WSK conceived the study and will lead the implementation of the trial. WSK and SHL participated in the study design and drafted the manuscript. WSK and NJP participated in the intervention design by developing specific protocols and helped with the implementation. SHL and JK will collect data and participate in the analysis. JP will lead the statistical analysis. All authors have read and approved the final manuscript. Two independent investigators blinded to the group assignment, SHP and NRK, will conduct the primary and secondary outcome assessments.

Won-Seok Kim orcid: 0000-0002-1199-5707.
